# Severe fever with thrombocytopenia syndrome virus: a systematic review and meta-analysis of transmission mode

**DOI:** 10.1017/S0950268820002290

**Published:** 2020-09-30

**Authors:** X. Y. Huang, Z. Q. He, B. H. Wang, K. Hu, Y. Li, W. S. Guo

**Affiliations:** 1 Henan Province Center for Disease Control and Prevention, Zhengzhou, China; 2 Henan Key Laboratory of Pathogenic Microorganisms, Zhengzhou, China; 3 College of Public Health, Zhengzhou University, Zhengzhou, China; 4 Henan Academy of Medical Sciences, Zhengzhou, China

**Keywords:** Meta-analysis, severe fever with thrombocytopenia syndrome virus, ticks, transmission mode

## Abstract

Severe fever with thrombocytopenia syndrome (SFTS) is a disease with a high case-fatality rate that is caused by infection with the SFTS virus (SFTSV). Five electronic databases were systematically searched to identify relevant articles published from 1 January 2011 to 1 December 2019. The pooled rates with 95% confidence interval (CI) were calculated by a fixed-effect or random-effect model analysis. The results showed that 92 articles were included in this meta-analysis. For the confirmed SFTS cases, the case-fatality rate was 0.15 (95% CI 0.11, 0.18). Two hundred and ninety-six of 1384 SFTS patients indicated that they had been bitten by ticks and the biting rate was 0.21 (95% CI 0.16, 0.26). The overall pooled seroprevalence of SFTSV antibodies among the healthy population was 0.04 (95% CI 0.03, 0.05). For the overall seroprevalence of SFTSV in animals, the seroprevalence of SFTSV was 0.25 (95% CI 0.20, 0.29). The infection rate of SFTSV in ticks was 0.08 (95% CI 0.05, 0.11). In conclusion, ticks can serve as transmitting vectors of SFTSVs and reservoir hosts. Animals can be infected by tick bites, and as a reservoir host, SFTSV circulates continuously between animals and ticks in nature. Humans are infected by tick bites and direct contact with patient secretions.

## Introduction

Severe fever with thrombocytopenia syndrome (SFTS) is an emerging infectious disease that is caused by SFTS virus (SFTSV) [1]. From 2010 to 2016, more than 5000 confirmed SFTS cases have been reported in at least 23 provinces of mainland China, and the case-fatality rate of SFTS infection was 5.3% [2, 3]. SFTS cases have also been reported in South Korea, Japan and Vietnam, and a similar disease has occurred in the USA [4–7]. Due to the heavy burden, lack of vaccines, effective therapies and high-fatality rates, the disease has become an important health issue.

Some SFTS patients had been bitten by ticks before the onset of illness [8]. Animals might be a reservoir host in the life cycle of SFTSV in nature [9]. SFTSV has been isolated successfully from some ticks [10]. Until now, the natural transmission mode of SFTSV among humans, hosts and vectors has remained unclear.

In this study, we systemically reviewed three aspects of SFTS: SFTS cases and asymptomatic infections (human level), seroprevalence and SFTSV infection rates in animals (animal level), and SFTSV positivity rate in ticks and vertical transmission among ticks (tick level). Thereafter, the incidence rate of SFTS in the healthy population, the positive ratio of anti-SFTSV antibodies in animals and the infection rate of SFTSV in ticks were calculated to study the transmission mode of SFTSV. This mode of transmission was beneficial for blocking the transmission route and reducing the incidence of SFTS.

## Materials and methods

This systematic review followed the guidelines provided in the Cochrane Collaboration and Preferred Reporting Items for Systematic Reviews and Meta-Analyses (PRISMA). The PRISMA checklist is included in the Supporting Information.

### Search strategy

An electronic search of the Chinese National Knowledge Infrastructure databases and the Wan Fang Data, PubMed, Web of Science and Embase databases was performed for all eligible papers (published from 1 January 2011 until 1 December 2019; English and Chinese publications) using a range of search strings ('severe fever with thrombocytopenia syndrome’ or ‘SFTS’ or ‘fever, thrombocytopenia and leukopenia syndrome’ or ‘FTLS’ or ‘severe fever with thrombocytopenia syndrome virus’ or ‘SFTSV’ or ‘fever, thrombocytopenia and leukopenia syndrome virus’ or ‘FTLSV’ or ‘Huaiyangshanvirus’ or ‘HYSV’ or ‘New bunyavirus’ or ‘NBV’ or ‘Dabie mountain virus’ or ‘DBMV’ or ‘Dabie bandavirus’ or ‘Huaiyangshan banyangvirus’ or ‘BHAV’). Additional studies obtained from the references of the original articles were also included.

### Eligibility criteria

The article had been accepted for publication with full text available and should meet one of the following conditions: (1) SFTS patients must be confirmed and baseline information could be extracted, (2) people who had an asymptomatic infection were confirmed by SFTSV antibodies (IgM and IgG), (3) animals were confirmed by SFTSV antibodies or RNA and (4) transmission medium was confirmed by SFTSV RNA. Exclusion criteria included abstracts, conferences, letters, reviews, duplicated publications, and overlapping data sets.

SFTS patients mentioned in the selected studies were confirmed as meeting one or more of the following criteria: (1) the virus was isolated from the patient's samples, (2) SFTSV RNA was detected in the patient's serum and (3) a fourfold or greater increase in antibody titres was detected between paired patient serum samples collected from the acute and convalescent phases of infection.

### Data extraction and quality assessment

For this meta-analysis, the following information was extracted from every eligible article: first author, year of publication, country, province; year of admitted patients, confirmed cases, death number, test method, and patients' age (SFTS patients); investigation time, sample size and the number of asymptomatic infected people with SFTSV (asymptomatic infections); sampling time and sample size of infected animals; sampling time of transmission medium and testing result.

The included studies were assessed using Study Quality Assessment Tools provided by the National Institute of Health [11], which consisted of good, fair and poor. Based on the quality assessment for studies, we evaluated the articles' quality.

### Statistical analysis

The statistical analyses were performed using SPSS 13.0 software (SPSS, Inc., Chicago, IL), R software (version 4.0.0), and STATA version 12.0 (STATA Corporation, College Station, Texas, USA) [12, 13]. Means and s.d. were calculated to describe continuous variables with normal distribution, medians and ranges or interquartiles were calculated to describe the abnormal distribution. Each study calculated the event rates and proportions with confidence limits by the R software package [12]. The *χ*^2^ test was used for comparison between groups, Cochran Q and I^2^ statistics were used to assess the heterogeneity among the studies [14]. A Cochran Q test with a *P*-value of <0.05 was considered to be statistically significant. An I^2^ value of more than 75% indicated high heterogeneity, and then a random-effect model was used. Otherwise, a fixed-effect model was performed. In some circles, researchers tended to start with the fixed-effect model, and then switched to the random-effects model if there was a compelling reason to do so [15]. Because of the high heterogeneity, a random effects model was more suitable when combining results from the studies. A leave-one-out sensitivity analysis was carried out to assess the impact of each study on the overall pooled estimate. Publication bias was appraised using Begg's test or Egger's test [16, 17]. A *P*-value of <0.05 was considered statistically significant.

## Results

### Literature search

**Fig. 1.**
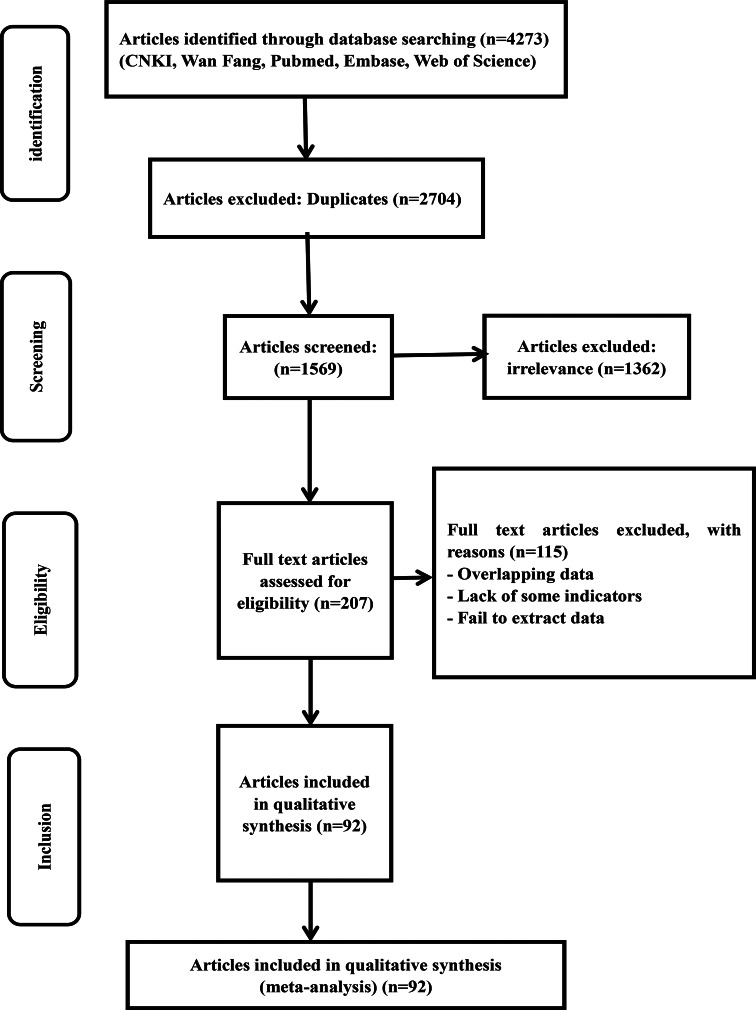
Flow chart of the study selection process in this meta-analysis.

A total of 4273 articles were retrieved through the database searches. A total of 2704 articles were excluded because they were duplicates and 1362 irrelevant studies were removed. Then, a total of 207 articles were evaluated for eligibility. A total of 115 studies were excluded for the following reasons: lack of some indicators, failure to extract data and overlapping data. For the same province, we selected documents with a large span of years and a large number of cases. After close scrutiny, 92 studies were included (Fig. 1).

### Study characteristics and quality assessment

The basic characteristics of the included studies and the quality assessment of the results are shown in Tables 1–5. These studies were published between 2011 and 2019 and carried out in three countries with different geographical locations. Sixty-nine studies were performed in China, 16 studies were conducted in South Korea and seven studies were conducted in Japan. The quality of the assessment result, based on the Study Quality Assessment Tools, is shown in 15 studies that were of high quality, 10 studies were of poor quality and 76 studies were of moderate quality.

**Table 1.** Basic characteristics of SFTS patients

First author	Publication year	Country	Province	Year of admitted patients	Age	Confirmed case	Death number	Test method	Language	Quality Rating
Bao *et al*. [18]	2011	China	JS	2007	61.4 (10.5)^a^	7	1	RT–PCR	English	Poor
Liu *et al*. [19]	2012	China	HB	2010	56 (11–81)^b^	87	8	RT–PCR	Chinese	Fair
Gai *et al*. [20]	2012	China	SD	2008.5–2011.7	61 (40–83)^b^	59	11	RT–PCR	English	Fair
Deng *et al*. [8]	2013	China	LN	2010.6–2011.12	55 (17–89)^b^	115	14	RT–PCR/ELISA	English	Good
Ding *et al*. [21]	2014	China	SD & HN	2010–2011	61 (23–82)^b^	59	7	RT–PCR	English	Fair
He *et al*. [22]	2014	China	HB	2010.5–2012.12	59 (36–86)^b^	73	19	RT–PCR	Chinese	Poor
Sun *et al*. [23]	2014	China	ZJ	2011–2013	66 (31–84)^b^	65	9	RT–PCR	English	Fair
Shin *et al*. [24]	2015	Korea	NA	2013.3	69 (28–84)^b^	35	16	RT–PCR	English	Fair
Wei *et al*. [25]	2015	China	SX	2013.6	66	1	0	RT–PCR	English	Fair
Xu *et al*. [26]	2015	China	HN	2007.4–2011.5	51.4 (13.0)	422	68	RT–PCR	Chinese	Fair
Choi *et al*. [27]	2016	Korea	NA	2013–2015	67.5 (57–76)^c^	172	56	RT–PCR/IFA	English	Good
Kato *et al*. [28]	2016	Japan	NA	2013.3–2014.9	78 (65–84)^c^	49	15	RT–PCR	English	Good
Zhao *et al*. [29]	2016	China	AH & JS	2010.10–2014.8	57.6 (38–78)^b^	40	7	RT–PCR	Chinese	Fair
Hu *et al*. [30]	2017	China	JS	2011–2013	56.5 (76–83)^b^	89	19	RT–PCR/ELISA	English	Fair
Wang *et al*. [31]	2017	China	HB	2011.1–2016.12	59.3 (23–87)^b^	521	44	RT–PCR/ELISA	English	Fair
Hu *et al*. [32]	2018	China	ZJ	2014.1–2017.4	57.8 (12.66)	25	5	RT–PCR/ELISA	English	Fair
Jia *et al*. [33]	2018	China	JS	2010.10–2016.10	59 (51–67)^c^	90	20	RT–PCR	English	Fair
Xia *et al*. [34]	2018	China	AH	2014.4–2017.12	62 (10.82)	86	12	RT–PCR/ELISA	Chinese	Fair
Xu *et al*. [35]	2018	China	SD	2014.1–2015.12	65.82 (11.36)	60	20	RT–PCR	English	Good
Song *et al*. [36]	2018	China	AH	2011–2017	64 (24–86)^c^	87	12	RT–PCR	Chinese	Poor
Li *et al*. [37]	2018	China	HN	2011.4–2017.10	61.4 (12.20)	2096	340	RT–PCR/ELISA	English	Good
Kwon *et al*. [38]	2018	Korea	Seoul	2015.7–2016.10	60 (7.00)	11	1	RT–PCR	English	Fair
Chen *et al*.[39]	2019	China	SD	2010–2017	NA	2731	251	RT–PCR/ELISA	English	Fair
He *et al*. [40]	2019	China	HN	2017.8–2018.8	63.76 (11.88)	74	0	RT–PCR	Chinese	Fair
Kim *et al*. [41]	2019	Korea	Jeju	2014.7–2018.11	NA	55	6	RT–PCR/ELISA	English	Fair
Takahashi *et al*. [42]	2019	Japan	NA	2015.11–2018.4	71.14 (10.35)	7	1	NA	English	Fair
Zong *et al*. [43]	2019	China	LN	2011–2017	NA	438	19	RT–PCR	Chinese	Fair

Abbreviations: NA, not available; JS, Jiangsu; HB, Hubei; SD, Shandong; LN, Liaoning; HN, Henan; ZJ, Zhejiang; SX, Shaanxi; AH, Anhui; RT–PCR, reverse transcription–polymerase chain reaction; IFA, immunofluorescence assay; ELISA, enzyme-linked immunosorbent assay.

a Values the mean (s.d.).

b Values are listed as median (ranges).

c Values are listed as median (interquartiles).

**Table 2.** Basic characteristics of person-to-person transmission

First author	Publication year	Country	Index patient			Secondary patients	Language	Test method
			Age	Sex	Occupation			
Bao *et al*. [18]	2011	China	80	Female	Farmer	Relatives	English	RT–PCR
Gai *et al*. [44]	2011	China	77	Male	Farmer	HCWs and relatives	English	ELISA
Liu *et al*. [45]	2012	China	50	Female	NA	HCWs and relatives	English	RT–PCR/IFA
	56	Female	Farmer	Relatives	English	RT–PCR/IFA		
Chen *et al*. [46]	2013	China	63	Male	NA	Relatives	English	RT–PCR
Tang *et al*. [47]	2013	China	58	Male	NA	HCWs and relatives	English	RT–PCR
Wang *et al*. [48]	2014	China	78	Male	NA	Relatives	English	RT–PCR
Gong *et al*. [49]	2015	China	66	Female	Farmer	HCWs and relatives and neighbours	English	RT–PCR
Jiang *et al*. [50]	2015	China	66	Female	Farmer	Relatives	English	RT–PCR/ ELISA
Kim *et al*. [51]	2015	Korea	68	Female	NA	HCWs	English	RT–PCR
Yoo *et al*. [52]	2016	Korea	74	Male	Cattle rancher	Relatives	English	RT–PCR
Huang *et al*. [53]	2017	China	65	Female	Farmer	HCWs and relatives and neighbours	English	RT–PCR
Moon *et al*. [54]	2018	Korea	57	Male	NA	HCWs	English	RT–PCR/IFA

Abbreviations: NA, not available; RT–PCR, reverse transcription–polymerase chain reaction; IFA, immunofluorescence assay; ELISA, enzyme-linked immunosorbent assay; HCW: health care worker.

**Table 3.** Characteristics of asymptomatic infected persons

First author	Publication year	Region	Sampling time	Sample size	Number of positive	Test method	Language	Quality rating
Zhang *et al*. [55]	2011	Jiangsu, China	2010.7–2010.11	1922	18	D-ELISA	Chinese	Fair
Jiao *et al*. [56]	2011	Jiangsu & Anhui China	2010	250	9	D-ELISA	English	Fair
Zhao *et al*. [57]	2012	Shandong, China	2011.6	237	2	D-ELISA	English	Fair
Cui *et al*. [58]	2013	Shandong, China	2011	78	1	D-ELISA	English	Fair
Niu *et al*. [59]	2013	Shandong, China	2011	2590	140	I-ELISA	Chinese	Good
Wang *et al*. [60]	2013	Shandong, China	2010–2011	315	4	D-ELISA	Chinese	Poor
Zhan *et al*. [61]	2013	Hubei, China	2010–2012	957	61	ELISA	Chinese	Poor
Li *et al*. [62]	2014	Jiangsu, China	2012.3–2013.1	2547	33	D-ELISA	English	Fair
Liang *et al*. [63]	2014	Jiangsu, China	2011	2510	10	D-ELISA	English	Fair
Zhang *et al*. [64]	2014	Zhejiang, China	2013.6	986	71	ELISA	English	Fair
Hu *et al*. [65]	2015	Henan, China	2011.7–2013.12	5245	343	ELISA	English	Fair
Wei *et al*. [25]	2015	Shaanxi, China	2014	363	20	D-ELISA	English	Fair
Sun *et al*. [66]	2015	Zhejiang, China	2013	1380	76	ELISA	English	Fair
Tan *et al*. [67]	2015	Jiangsu, China	2010–2011	866	2	D-ELISA	Chinese	Fair
Xu *et al*. [68]	2015	Anhui, China	2013.9–2013.10	166	14	ELISA	Chinese	Fair
Zhou *et al*. [69]	2015	Shandong, China	2011.4–2011.12	237	2	D-ELISA	Chinese	Fair
Huang *et al*. [70]	2016	Anhui, China	2012.6	270	17	ELISA	English	Fair
Luo *et al*. [71]	2016	Shandong, China	2015.11–2016.1	628	33	ELISA	Chinese	Good
Xing *et al*. [72]	2016	Hubei, China	2012.8–2013.5	419	35	D-ELISA	English	Good
Lyu *et al*. [73]	2016	Anhui, China	2014–2015	2126	99	ELISA	English	Fair
Kim *et al*. [74]	2017	Busan, Korea	2015.5	1069	22	D-ELISA	English	Fair
Gokuden *et al*. [75]	2018	Kagoshima, Japan	2015.7–2016.1	646	2	ELISA	English	Fair
Kimura *et al*. [76]	2018	Ehime, Japan	2015.7–2015.8	694	8	ELISA	English	Fair
Shen *et al*. [77]	2019	Zhejiang, China	2018.6	439	13	D-ELISA	English	Fair
Du *et al*. [78]	2019	Henan, China	2016.4–2016.5	1463	165	I-ELISA	English	Fair

Abbreviations: ELISA, enzyme-linked immunosorbent assay; D-ELISA, double-antigen sandwich enzyme-linked immunosorbent assay; I-ELISA, indirect enzyme-linked immunosorbent assay.

**Table 4.** SFTSV seroprevalence in animals

First author	Publication year	Country	Sampling time	Sample size	No. of infected animals	Prevalence (%)	Test method	Language	Quality rating
Zhang *et al*. [55]	2011	China	2010.7–2010.11	931	103	11.06	D-ELISA	Chinese	Fair
Jiang *et al*. [79]	2012	China	2010.9	106	34	32.08	D-ELISA	Chinese	Good
Liu *et al*. [19]	2012	China	2010	19	12	63.16	D-ELISA	Chinese	Fair
Zhao *et al*. [80]	2012	China	2011.6	134	111	82.84	D-ELISA	English	Fair
Cui *et al*. [58]	2013	China	2011.6–2012.12	78	20	25.64	ELISA	English	Fair
Ding *et al*. [81]	2013	China	2011	641	268	41.81	D-ELISA	English	Fair
Liu *et al*. [82]	2013	China	2009–2011	103	20	19.42	IFA	Chinese	Fair
Niu *et al*. [83]	2013	China	2011.4–2011.12	3039	1249	41.10	D-ELISA	English	Fair
He *et al*. [22]	2014	China	2010.5–2012.12	31	12	38.71	D-ELISA	Chinese	Poor
Li *et al*. [62]	2014	China	2012.3–2013.2	2741	335	12.22	D-ELISA	English	Fair
Liu *et al*. [84]	2014	China	2013.1–2013.8	775	9	1.19	D-ELISA	English	Fair
Du *et al*. [85]	2014	China	2012.7–2012.10	312	141	45.19	ELISA	Chinese	Fair
Xu *et al*. [86]	2014	China	2010–2011	452	9	1.99	D-ELISA	Chinese	Fair
Tan *et al*. [67]	2015	China	2010–2011	215	6	2.79	D-ELISA	Chinese	Fair
Xu *et al*. [68]	2015	China	2013.9–2013.10	205	97	47.37	ELISA	Chinese	Fair
Li *et al*. [87]	2016	China	2013–2014	823	47	5.71	ELISA	English	Fair
Oh *et al*. [88]	2016	Korea	2013.5–2013.8	91	6	6.59	IFA	English	Fair
Xing *et al*. [72]	2016	China	2012.8–2013.5	50	27	54	ELISA	English	Good
Tabara *et al*. [89]	2016	Japan	2014.6–2015.3	510	11	2.16	ELISA	English	Fair
Hayasaka *et al*. [90]	2016	Japan	2006–2012	190	35	18.42	ELISA	English	Fair
Sun *et al*. [91]	2017	China	2014、2016	14	9	64	ELISA	English	Fair
Wang *et al*. [92]	2017	China	2015–2016	178	15	8.43	D-ELISA	English	Fair
Zhu *et al*. [93]	2017	China	2012–2014	354	0	0	ELISA	Chinese	Poor
Lee *et al*. [94]	2018	Korea	2016.3–2016.11	426	59	13.85	IFA	English	Good
Kimura *et al*. [76]	2018	Japan	2013.12–2014.2	107	20	18.69	ELISA	English	Fair
Kang *et al*. [95]	2018	Korea	2014–2015	737	43	6.89 (43/624)	D-ELISA	English	Fair
Yu *et al*. [96]	2018	Korea	2017.3–2017.8	207	30	14.49	ELISA	English	Fair
Huang *et al*. [9]	2019	China	2016.5–2018.4	615	275	44,72	ELISA	English	Fair
Yu *et al*. [97]	2019	Korea	2015–2017	215	40	18.60	I-ELISA	English	Fair
Yang *et al*. [98]	2019	China	2016.5–2016.6	1097	521	47.49	I-IFA	English	Fair

Abbreviations: ELISA, enzyme-linked immunosorbent assay; D-ELISA, double-antigen sandwich enzyme-linked immunosorbent assay; I-ELISA, indirect enzyme-linked immunosorbent assay; IFA, immunofluorescence assay; I-IFA, indirect immunofluorescence assay.

**Table 5.** SFTSV tick infections rates and vertical transmission characteristics

First author	Publication year	Country	Sampling time	No. of ticks	No. of infected pools	No. of tick pools	Language	Quality rating
Zhang *et al*. [99]	2011	China	2010.7–2010.10	3498	18	365	English	Fair
Yun *et al*. [100]	2014	Korea	2013.5–2013.10	212	12	148	English	Fair
Hayasaka *et al*. [101]	2015	Japan	2013.5–2013.8	1709	0	57	English	Fair
Luo *et al*. [10]	2015	China	2013.6–2013.7	3300	25	73	English	Fair
Wang *et al*. [102]	2015	China	2011	3048	122	1952	English	Fair
Li *et al*. [87]	2016	China	2013–2014	8520	45	722	English	Fair
Oh *et al*. [88]	2016	Korea	2013.5–2013.8	667	27	293	English	Fair
Yun *et al*. [103]	2016	Korea	2014.3–2014.10	17 570	5	23	English	Good
Tian *et al*. [104]	2017	China	2013.7–2013.9	11	0	2	English	Fair
Zhu *et al*. [93]	2017	China	2012–2014	113	0	64	Chinese	Poor
Zhuang *et al*. [105]	2018	China	2011	4910	89	202	English	Good
Jung *et al*. [106]	2018	Korea	2015–2017	3880	0	281	English	Fair
Yang *et al*. [98]	2019	China	2016.5–2016.6	4595	3	416	English	Fair

### SFTS cases and asymptomatic infections

#### Epidemiology of SFTS patients

**Fig. 2.**
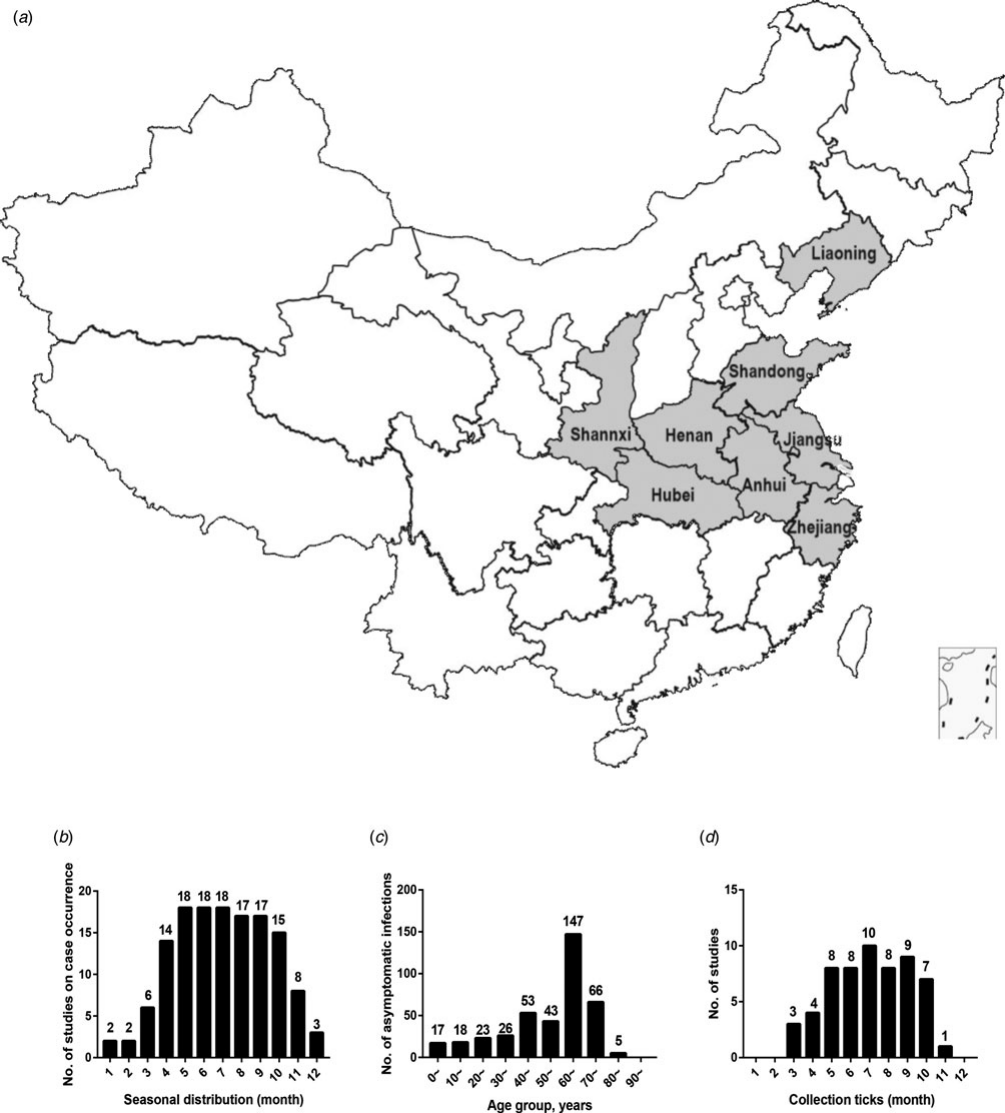
(a) Geographic distribution of SFTS in mainland China. (b) Seasonal distribution of published studies on case occurrence. (c) Age distribution of asymptomatic infections. (d) The relationships between collected ticks and number of published studies. The horizontal ordinate represented the month and the ordinate represented the number of studies that meet the requirements (b and d). The horizontal ordinate represented the age group and the ordinate represents the number of asymptomatic infections (c).

**Fig. 3.**
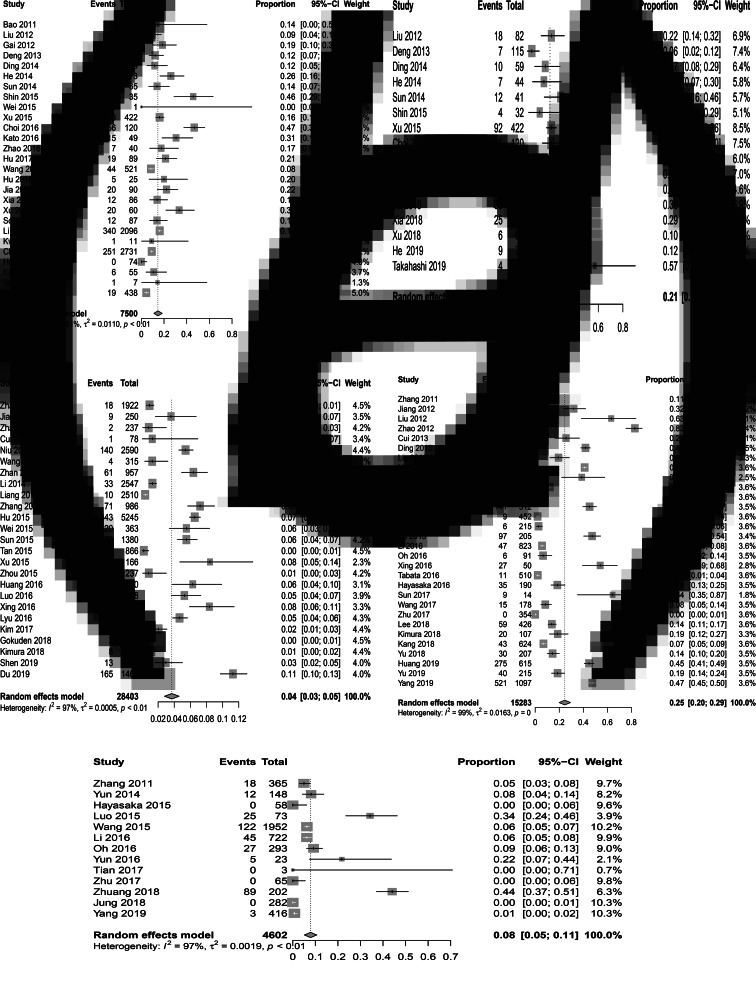
Forest plots of the meta-analysis on a panel of prevalence. (a) The pooled case-fatality rate of SFTS. (b) The pooled biting rate by ticks. (c) The overall seroprevalence of SFTSV among the healthy population. (d) The overall seroprevalence of total antibodies against SFTSV in animals. (e) Infection rate of SFTSV in ticks.

As shown in Table 1, 27 studies were included in the meta-analysis. A total of 7554 confirmed cases were collected from these studies. The geographical distribution was mainly in China (Zhejiang, Liaoning, Henan, Shandong, Jiangsu, Anhui, Hubei province), Japan and South Korea (Fig. 2A). SFTS showed strong seasonality, the cases were mainly reported from April to October and peaked between May and July, and cases during those three months accounted for 54.39% (3792/6972) of all cases (Fig. 2B). Of the 7409 cases, 47.52% (3521/7409) were male and 52.48% (3888/7409) were female. The median age of the patients was 61 years old (range: 11–89). The case-fatality rate was 0.15 (95% CI 0.11, 0.18) and the vast majority of the cases were farmers (82.89%, 4307/5196), including agricultural and forest workers living in rural areas (Fig. 3A). A total of 296 of 1384 SFTS patients indicated that they had been bitten by ticks, the biting rate was 0.21 (95% CI 0.16, 0.26), and the result showed statistically significant heterogeneity (*I*^2^ = 77.0%, *P* < 0.001) (Fig. 3B).

#### Person-to-person transmission

Person-to-person transmission of SFTSV was mostly reported in hospitals from China, Japan or South Korea. According to epidemiological investigations and laboratory analyses, 12 studies reported that SFTSV could be transmitted from person to person by contact with blood or bloody respiratory secretions, especially in inadequately protected people (Table 2). There were 84 secondary patients, but only three tertiary patients were described or found in articles. All 27 studies with 7554 confirmed patients tried to find the index cases but did not describe them in these articles.

#### Asymptomatic infections

The 28 403 blood samples in 25 studies from 2011 to 2019 were collected from healthy people. These were a random sample of healthy people from some populations in each study. Through collecting serum of healthy people and testing their IgM and IgG, we could calculate the overall pooled seroprevalence of SFTSV antibodies among the healthy population. Based on the data extractability, 24 075 healthy people, including 11 647 males and 12 428 females (male to female ratio: 0.94:1), were extracted from 17 studies. Among healthy people (24 075), 5059 had clear occupational descriptions in these studies and 3999 (79.05%) of them were farmers (Table 3).

A total of 931 healthy people were tested positive for SFTSV antibodies, 449 (48.23%) cases were male and 482 (51.77%) cases were female, there was no significant difference between the male and female groups (*t* = −0.202, *P* = 0.84). The participants were grouped by decades and the results showed that a large number of asymptomatic infections were 60–70 years (Fig. 2C). In addition, the overall pooled seroprevalence of SFTSV antibodies among the healthy population in the random-effect model was 0.04 (95% CI 0.03, 0.05) (Fig. 3C).

### Seroprevalence and SFTSV infection rates in animals

#### The seroprevalence of SFTSV in animals

We analysed 30 studies to determine the overall seroprevalence of SFTSV in animals (Table 4). These included studies that did not describe the sampling method but just described that animal serums were collected at the survey site (there were SFTS cases nearby) for laboratory testing of IgM and IgG. The overall seroprevalence of SFTSV in animals was 0.25 (95% CI 0.20, 0.29) and is displayed as a forest plot in Figure 3D. The goats and sheep were 0.49 (95% CI 0.34, 0.65) and 0.42 (95% CI 0.31, 0.57) in cattle, 0.16 (95% CI 0.10, 0.22) in chickens, 0.26 (95% CI 0.17, 0.35) in dogs and 0.04 (95% CI 0.01, 0.07) in pigs. The *χ*^2^ test was used for comparison of the seroprevalence in different animals and there was a statistically significant difference in the positivity rates of these animals (*χ*^2^ = 1204.92, *P* < 0.001). The seroprevalences of SFTSV in goats and cattle were higher than those in animals.

#### The positivity rates of SFTSV RNA in animals

In our included studies, RT–PCR was used to detect SFTSV RNA in these animals. Fourteen articles reported that the infection rates of SFTSV RNA in animals ranged from 0.23 to 26.31%. The positivity rates of SFTSV RNA detected in sheep and goats were 0.03 (95% CI 0.01, 0.04), 0.04 (95% CI 0.01, 0.08) in cattle, 0.02 in chickens, 0.03 in dogs, 0.02 in pigs, 0.02 (95% CI 0.01, 0.04) in shrews, 0.02 in yellow weasels, 0.03 in hares, 0.01 (95% CI 0.00, 0.02) in deer and 0.02 (95% CI 0.01, 0.03) in rodents. There were statistically significant differences in the positivity rates of different animals (*χ*^2^ = 31.97, *P* < 0.001), and the positivity rate of SFTSV RNA in cattle was higher than that in goats (*χ*^2^ = 4.49, *P* = 0.03). Twenty-three strains of the virus were isolated from animal specimens in eight articles, including cattle, dogs, rodents, shrews, water deer, wild boars, goats, sheep, yellow weasels, and hedgehogs.

### SFTSV positivity rate in ticks and vertical transmission

#### The infection rate of SFTSV in ticks

A total of 4598 ticks were included in 13 studies. These included studies that did not describe the sampling method but just described that ticks were collected at the survey site (there were SFTS cases nearby) for laboratory testing of SFTSV RNA. SFTSV RNA was detected in 346 ticks via laboratory tests and we found that there were not positive numbers in four articles (Table 5). The infection rate of SFTSV in ticks was 0.08 (95% CI 0.05, 0.11) and the result had significant heterogeneity (*I*^2^ = 97.0%, *P* < 0.001) (Fig. 3E). There were 65 strains of SFTSV isolated from ticks. The seasonal change in tick numbers was consistent with SFTS case reports (Fig. 2D).

#### The positivity rate of SFTSV in vertical transmission

In vertical transmission, the experiment of ticks in the laboratory and the detection of naturally infected oviposited ticks were reported in previously published articles. Luo *et al*. reported that ticks fed on SFTSV-infected mice could acquire the virus and transovarially transmit it to other developmental stages of ticks. Wang *et al*. reported that 2 of 22 egg masses oviposited by blood-fed *Haemaphysalis longicornis* females were positive for SFTSV [102]. Three studies containing 168 ticks were analysed. A total of 117 ticks (eggs, larvae, nymphs or adult ticks) were infected through female tick oviposits. The positivity rate of SFTSV was 0.55 (95% CI: 0.12, 0.97) and the heterogeneity was also statistically significant (*I*^2^ = 97.3%, *P* < 0.001). For the other transmission media, SFTSV has been detected by RT–PCR in gamasid mites, chigger mites and gadflies. The gamasid mites and chigger mites in the nine groups were all positive for SFTSV genomic nucleic acids. A total of 38 gadflies were divided into 16 groups and the results showed that three groups of gadflies were positive by RT–PCR. The role of other bloodsucking insects such as vectors and reservoirs for SFTSV needs to be further investigated [107, 108].

#### Virus isolation and phylogenetic analysis

**Fig. 4.**
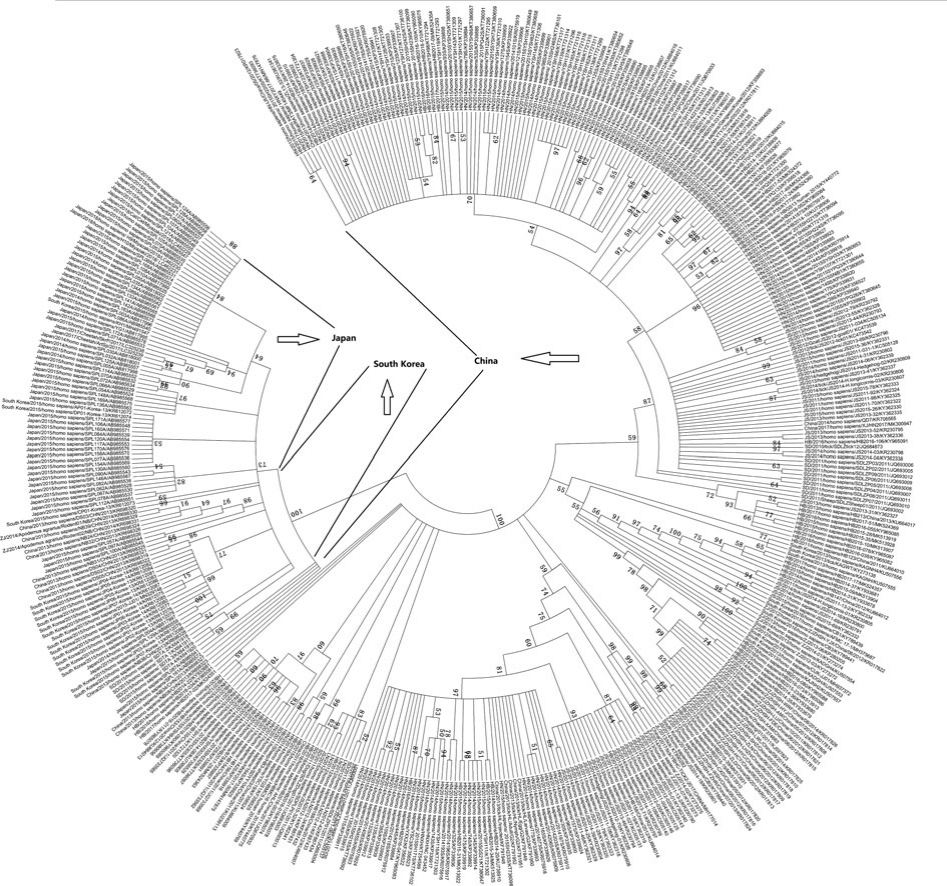
Phylogenetic analysis of the S segment of 445 SFTSV complete sequences obtained from GenBank.

Eight articles reported that virus isolation was attempted on all viral RNA-positive serum samples, and phylogenetic analysis of the S segment of these SFTSV isolates was performed. For further analysis, the S segment of 445 SFTSV complete sequences obtained from GenBank was analysed, and we found that most of the viral isolates from animals and ticks were genetically close to the SFTS patient-derived isolates, and there was a clear boundary in these isolates in the three countries (Fig. 4). Niu *et al*. also showed that all sequences of the isolates from domesticated animals, SFTS patients and *H. longicornis* ticks shared more than 95% identity, which demonstrated a close evolutionary relationship among those SFTSV isolates from domesticated animals, ticks and SFTS patients by pairwise distance analysis [83].

#### Subgroup analysis

To explore the potential sources of high heterogeneity in the meta-analysis, we performed a subgroup analysis by country. The pooled case-fatality rate of SFTS in China was 0.13 (95% CI 0.10, 0.17) (*I*^2^ = 91.0%, *P* < 0.01), 0.29 in Japan (95% CI 0.18, 0.42) (*I*^2^ = 00.0%, *P* = 0.39), and 0.26 in South Korea (95% CI 0.11, 0.50) (*I*^2^ = 86.0%, *P* < 0.01). The more details with other prevalences are presented in Supplementary Table S1.

#### Sensitivity analysis and publication bias

The sensitivity analysis was performed, which indicated little change in the data (Supplementary Table S2). The incidence rate of SFTS in the sensitivity analysis was stable and had no significant effect on the merger rate.

Egger's test and Begg's test were conducted to evaluate publication bias. The results showed that Egger's test *t* value was 2.76 (*P* = 0.011), and Begg's test *z* value was 1.77 (*P* = 0.076) in the case-fatality rate of SFTS. Egger's test *t* value was 4.44 (*P* = 0.000) and Begg's test *z* value was 2.15 (*P* = 0.032) in the overall seroprevalence of SFTSV among the healthy population. Egger's test *t* value was 3.52 (*P* = 0.002) and Begg's test *z* value was 2.27 (*P* = 0.023) in the seroprevalence of SFTSV in animals. Egger's test *t* value was 3.23 (*P* = 0.008) and Begg's test *z* value was 0.92 (*P* = 0.360) in the infection rate of SFTSV in ticks.

## Discussion

This systematic review and meta-analysis were performed to study the transmission mode of SFTSV. The epidemiology of SFTS cases has the following characteristics: (1) most patients were older (60–70 years), (2) May to July was the peak of the SFTS cases in these epidemic areas and (3) most of the reported cases were farmers living or working in wooded and hilly areas, where ticks were commonly found. The epidemic areas of SFTS were mainly in the central and eastern China, mostly in mountainous and hilly rural areas, while there was no case in the western region, which might be due to the topography of mountains and plateaus. Infection and death cases were mainly found in central China, where *H. longicornis* ticks were spread [3]. For the person-to-person transmission of SFTSV, we discovered the index and secondary patients but only three tertiary patients were described or found. The index cases died soon after becoming infected, suggesting that their transmission rate might be low. The asymptomatic infection rate was calculated and was high among the healthy population. This result was similar to a previously reported study [109]. These observations indicated that the rate of asymptomatic infection increased with the SFTS epidemic situation.

Animal hosts and vectors of SFTSV are still unclear, but some case-control studies have reported that raising animals is a risk factor for human SFTS and working and living with domesticated animals, especially those showing high levels of SFTSV antibodies, increases SFTS incidence rates [110, 111]. It is possible that SFTSV could be transmitted directly from animals (other than ticks) to humans through contact with blood and/or other body fluids. However, there are few articles at present, and we could not perform further research. The seroprevalence of SFTSV in animals was conducted in previous studies. Du and Chen *et al*. investigated whether SFTSV has a wide spectrum of animal hosts, including domestic and wild animals. The prevalence of SFTSV is high among specific animal species [78, 111]. In our study, we searched and analysed the publications, and the results showed that the overall seroprevalence of SFTSV in animals was 25%. Previous studies reported that the sequences of SFTSV isolated from animals were highly homologous to SFTSV from human cases [9, 83, 88]. The included study also showed that the virus was isolated from animals, such as cattle, goats and hedgehogs. A natural infection study also showed that goats were infected by ticks in the SFTS-endemic region. The goats were viraemic over a very short period (<24 h) after the viral infection and soon occupied by a timely mounting antibody response that effectively controlled the infection. The whole cohort did not show any specific clinical signs of illness and all survived infection [112].

Ticks, especially *H. longicornis*, are suspected to be potential vectors and have a broad animal host range in nature [113]. The positivity rate of SFTSV indicated that SFTSV could be carried by ticks and transmitted vertically through female tick oviposits. Luo *et al*. fed *H. longicornis* ticks to SFTSV-infected mice, and the results indicated that ticks could acquire SFTSV from infected mice. The team also fed SFTSV-infected ticks on Kunming albino mice, and the results showed that ticks transmitted SFTSV to mice through feeding. These results from a laboratory study confirmed that ticks could serve as a vector and reservoir of SFTSV and were consistent with those of epidemiological investigations [10]. A previous study reported that the prevalence of SFTSV infection among ticks collected from vegetation was lower than that among ticks collected from animals. Ticks were vectors of SFTSV, similar to other insect-borne diseases, and SFTSV could not spread among ticks except for vertical transmission, so the SFTSV infection rate of vegetated ticks was fairly low [102]. Although the SFTSV of mites and gadflies was detected, we did not have much evidence that they were the main routes of transmission.

The case-fatality rate of SFTS has a wide range among endemic areas, the reason might be that SFTS cases were first found in China, the case-fatality was high at first and then gradually decreased. Later, Japan and South Korea successively reported cases, which led to a large range. The overall seroprevalence of SFTSV among the healthy population was almost the same in the three countries. The seroprevalence of SFTSV in animals has varied widely among the three countries, the reason for this discrepancy might include different geographical locations. In this study, we found SFTSV seroprevalence was high in China and was relatively low in Japan. However, this result should be interpreted with caution because of the limited number of studies and sample size in Japan and South Korea, which might lead to a lack of representativeness. Because only one study regarding Japan was included, we could not perform further analysis in the infection rate of SFTSV in ticks.

Our study had several strengths. The poor, moderate and high-quality studies were pooled for a relatively large sample size. Through the analysis of previous studies, we summarised the transmission mode of SFTSV, which had guiding significance for cutting off the transmission channels. Nevertheless, this meta-analysis also had some limitations. First, significant heterogeneity brought into question the suitability of performing this meta-analysis. Second, publication bias might distort the estimates of rates, so the results should be interpreted with caution.

## Conclusion

**Fig. 5.**
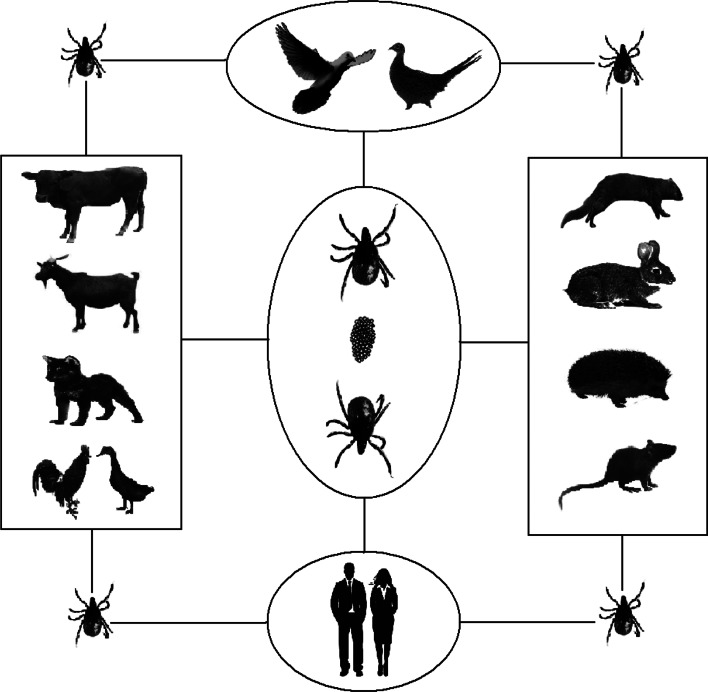
Transmission models of SFTSV among ticks, animals and humans.

According to the current study, the transmission patterns of SFTSVs can be summarised as shown in Figure 5. Ticks can serve as transmitting vectors of SFTSV and act as reservoir hosts as well. Animals can be infected with SFTSV by tick bites and as a reservoir host, but most animals are latent, and the infected animals might introduce SFTSV into areas where ticks were present. SFTSV circulates continuously between animals and ticks in nature. Humans are infected by SFTSV-infected ticks and might be infected by direct contact with infected blood or body fluids of patients.

## Data Availability

The datasets used and/or analysed during the current study are available from the corresponding author upon reasonable request.

## Supplementary material

For supplementary material accompanying this paper visit https://doi.org/10.1017/S0950268820002290. click here to view supplementary material
