# Supplementary material for: Severe fever with thrombocytopenia syndrome virus: a systematic review and meta-analysis of transmission mode
Source: Epidemiol Infect. 2020 Sep 30;148:e239. doi: 10.1017/S0950268820002290 (PMC7584033; doi:10.1017/S0950268820002290)
Supplement: Supplementary file 1 [file S0950268820002290sup.zip › S0950268820002290sup001.docx]

**Epidemiology and Infection**

**Severe fever with thrombocytopenia syndrome virus: a systematic review and meta-analysis of transmission mode**

X.Y. Huang^1,2^, Z.Q. He^3^, B.H. Wang^3^, K. Hu^4^, Y. Li^1,2^ and W.S. Guo^1#^

**Supplementary Material:**

**Table S2** Sensitivity analysis in this review.

| Groups | No. Studies. | Meta-analysis, pooled rate (95% CI) | Heterogeneity, P-value (I-squared) | Sensitive analysis (remove single case data set) | Heterogeneity, P-value (I-squared) |
| --- | --- | --- | --- | --- | --- |
| **A** | 27 | 0.15 (0.11-0.18) | <0.01 (91.0%) | 0.17 (0.13-0.22) | <0.01 (92.5%) |
| **B** | 16 | 0.21 (0.16-0.26) | <0.01 (77.0%) | 0.22 (0.18-0.27) | <0.01 (75.1%) |
| **C** | 25 | 0.04 (0.03-0.05) | <0.01 (97.0%) | 0.03 (0.02-0.04) | <0.01 (94.4%) |
| **D** | 30 | 0.25 (0.20-0.29) | <0.01 (99.0%) | 0.18 (0.14-0.23) | <0.01 (98.6%) |
| **E** | 13 | 0.08 (0.05-0.11) | <0.01 (97.0%) | 0.07 (0.04-0.14) | <0.01 (97.2%) |

Abbreviations: A, the pooled case-fatality rate of SFTS; B, the pooled biting rate by ticks; C, the overall seroprevalence of SFTSV among the healthy population; D, the overall seroprevalence of total antibodies against SFTSV in animals; E, infection rate of SFTSV in ticks.
