# Supplementary material for: Severe fever with thrombocytopenia syndrome virus: a systematic review and meta-analysis of transmission mode
Source: Epidemiol Infect. 2020 Sep 30;148:e239. doi: 10.1017/S0950268820002290 (PMC7584033; doi:10.1017/S0950268820002290)
Supplement: Supplementary file 1 [file S0950268820002290sup.zip › S0950268820002290sup002.docx]

**Epidemiology and Infection**

**Severe fever with thrombocytopenia syndrome virus: a systematic review and meta-analysis of transmission mode**

X.Y. Huang^1,2^, Z.Q. He^3^, B.H. Wang^3^, K. Hu^4^, Y. Li^1,2^ and W.S. Guo^1#^

**Supplementary Material:**

**Table S1** Subgroup analysis by country in the meta-analysis.

| Groups | No. studies | Prevalence | Heterogeneity | Model |
| --- | --- | --- | --- | --- |
|  |  | P 95% CI | P^a^ I^2^ (%) |  |
| **The pooled case-fatality rate of SFTS** | | | | |
| China | 21 | 0.13 0.10-0.17 | <0.01 91 | R |
| South Korea | 4 | 0.26 0.11-0.50 | <0.01 86 | R |
| Japan | 2 | 0.29 0.18-0.42 | 0.39 0 | F |
| **The pooled biting rate by ticks** | | | | |
| China | 12 | 0.19 0.14-0.25 | <0.01 77 | R |
| South Korea | 2 | 0.20 0.14-0.27 | 0.25 0 | F |
| Japan | 2 | 0.45 0.32-0.58 | 0.48 0 | F |
| **The overall seroprevalence of SFTSV among the healthy population** | | | | |
| China | 22 | 0.03 0.02-0.05 | <0.01 98 | R |
| South Korea | 1 | 0.02 0.01-0.03 | NA NA | NA |
| Japan | 2 | 0.01 0.00-0.02 | 0.09 31 | F |
| **The overall seroprevalence of total antibodies against SFTSV in animals** | | | | |
| China | 22 | 0.21 0.11-0.36 | <0.01 100 | R |
| South Korea | 5 | 0.12 0.08-0.16 | <0.01 83 | R |
| Japan | 3 | 0.09 0.03-0.27 | <0.01 95 | R |
| **Infection rate of SFTSV in ticks** | | | | |
| China | 8 | 0.06 0.02-0.18 | <0.01 99 | R |
| South Korea | 4 | 0.04 0.01-0.26 | <0.01 97 | R |
| Japan | 1 | 0.00 0.00-0.06 | NA NA | NA |

Abbreviations: NA, not available; F, fixed model; R, random model; P^a^, P value of Q-test for heterogeneity test.
